# The Predominance of *Klebsiella aerogenes* among Carbapenem-Resistant *Enterobacteriaceae* Infections in Japan

**DOI:** 10.3390/pathogens11070722

**Published:** 2022-06-24

**Authors:** Kosuke Kamio, J. Luis Espinoza

**Affiliations:** Faculty of Health Sciences, Kanazawa University, Kanazawa 920-1192, Japan; koooo.201@stu.kanazawa-u.ac.jp

**Keywords:** *Klebsiella aerogenes*, carbapenem-resistant *Enterobacteriaceae*, multidrug resistant organisms, carbapenem resistance, carbapenemase production, infection surveillance, *E. coli*, *Klebsiella pneumoniae*

## Abstract

The emergence of carbapenem-resistant Enterobacteriaceae (CRE) is an important public health issue worldwide, not only due to the potential of these pathogens for widespread dissemination, but also due to the limited antimicrobial therapy options, and the elevated mortality rates associated with these infections. As with other multidrug-resistant organisms (MDROs), active surveillance via timely testing, early diagnosis, and contact isolation is an important strategy to control the occurrence and spread of CRE bacteria. Here we summarize the epidemiology of CRE infections in Japan from 2015 to 2019. Data were extracted from a public dataset collected by the nationwide surveillance system via the National Institute of Infectious Diseases (NIID) of Japan. The annual number of reported CRE infections has remained relatively stable, with a tendency to increase in the last two years (1671 cases reported in 2015 and 2333 cases reported in 2019). The majority of patients who presented CRE infections over this five year period were older than 65 years (~80%, mean age 75), 60% of them were men, and mortality rates were around 3.5%. Importantly, about 60% of infections are caused by both *Enterobacter cloacae* and *Klebsiella aerogenes* (previously known as *Enterobacter aerogenes*), the former being the most common pathogen in 2015 and 2016 (~30%), and the latter the leading pathogen since 2017 (~40%). The most common carbapenemase isolated was the IMP carbapenemase type. Further studies are needed to determine the prevalence of CRE colonization, especially in the healthcare setting, and to elucidate the mechanisms behind the local predominance of *Klebsiella aerogenes* and *Enterobacter cloacae*.

## 1. Introduction

*Enterobacteriaceae* are a family of gram-negative bacteria that include beneficial commensals and highly virulent pathogens, such as *Escherichia coli*, *Klebsiella*, *Salmonella*, and *Shigella*. The emergence of pathogenic *Enterobacteriaceae* with acquired antibiotic resistance poses a serious threat to public health [1], as illustrated by the rise of *Enterobacteriaceae* strains with intrinsic resistance to ß-lactam antibiotics via the production of extended-spectrum beta-lactamases (ESBLs) in the past decades, which led to the extensive use of carbapenems as a first-line empirical antibiotic therapy in the clinical setting and eventually the emergence and propagation of carbapenem-resistant *Enterobacteriaceae* (CRE) strains with increased ability to spread in healthcare settings [2]. Furthermore, CREs are pathogens with acquired resistance to carbapenems antibiotics that can cause a variety of clinical infections, including pneumonia, urinary tract infections (UTI), sepsis, soft tissue infections, bloodstream infections (BSI), and others. In addition to their acquired resistance to carbapenems, CREs are often resistant to multiple antibiotics, including broad-spectrum β-lactams, fluoroquinolones, and aminoglycosides, and, thus, infections caused by these pathogens are difficult to treat. Importantly, although CRE infections most often occur in the healthcare setting, infections by these pathogens can be also seen in the community setting, even affecting healthy individuals [2,3].

Resistance to carbapenems in *Enterobacteriaceae* can be mediated by three recognized mechanisms, as follows: (1) the production of carbapenemases (the main mechanism), (2) the use of efflux pumps for the extrusion of carbapenem antibiotics, and (3) the loss of porin pore proteins in the outer membrane of gram-negative bacteria that impairs the permeability of carbapenem antibiotics [3]. Carbapenemases are β-lactamases capable of hydrolyzing penicillins, cephalosporins, monobactams, and carbapenems that, based on amino acid homology can be categorized into four major classes, labeled A, B, C, and D. Molecular classes A, C, and D include a serine-based hydrolytic mechanism at their active site, whereas class B enzymes are metalloenzymes that require zinc as a metal cofactor for their catalytic activities [3].

The management of CRE infections is usually made on a case-by-case basis, and typically involves the administration of combinations of antimicrobial agents, such as polymyxins, tigecycline, fosfomycin, and aminoglycosides [4]. In addition, novel β-lactam–β-lactamase inhibitor combination therapies appear to be promising therapeutic options, although the isolation of strains with specific resistance mechanisms to these novel agents has also been reported [5]. More recently, the use of fecal microbiota transplantation (FTM) has been also utilized with promising results [6]. 

In Japan, in September 2014, CRE infections were added to the list of category V of communicable infectious diseases, based on the Infectious Diseases Control Law. Under this national surveillance system, only cases with active infection caused by *Enterobacteriaceae* pathogens that meet the defined criteria of carbapenem resistance (minimum inhibitory concentration (MIC) for meropenem ≥2 μg/mL, or imipenem ≥2 μg/mL, and cefmetazole ≥64 μg/mL) are eligible for notification. However, cases with confirmed colonization in the absence of clinical symptoms are not reported (https://www.niid.go.jp/niid/ja/diseases/ka/cre.html. Japanese version, last accessed on 29 May 2022).

We aimed to delineate the key epidemiological characteristics of CRE infection in Japan, in an attempt to identify clinical and microbiological features suitable for clinical public health interventions. Here, we present the main epidemiological features of CRE infections in Japan over a five year period (2015 to 2019). Our data reveal particularly distinctive features, especially the predominance of *Klebsiella aerogenes* and *Enterobacter cloacae* as the most commonly isolated CRE bacteria, the finding that IMP is the predominant carbapenemase detected, and a relatively low mortality rate (<5%) reported among CRE infected patients. 

## 2. Results

### 2.1. Epidemiological and Clinical Characteristics of CRE Infections in Japan

**Figure 1:**
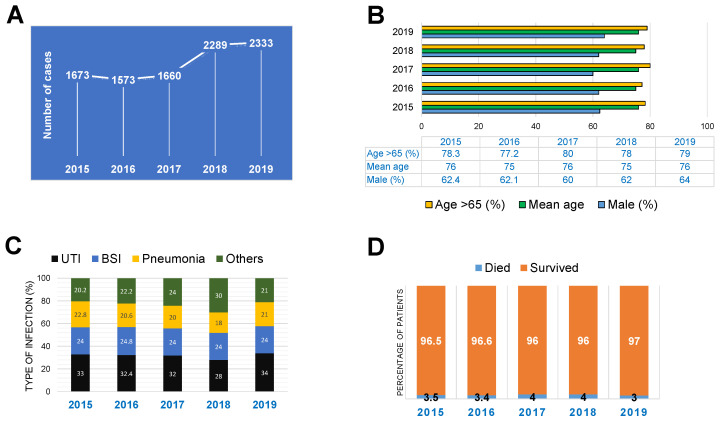
Key epidemiological findings. (**A**) The annual incidence of CRE infections. (**B**) Key demographic characteristics of the patients with CRE infections. (**C**) Type of infections where the CRE pathogens were isolated. (**D**) The annual survival rate of the patients with CRE infections.

The average number of annually notified CRE infections in 2015, 2016, and 2017 was approximately 1600, with a relatively stable CRE detection rate. The number of notified CRE infections increased in 2018 and 2019 with more than 2000 cases notified per year (Figure 1A). The majority of patients who presented CRE infections over this five year period were older than 65 years (~80%, mean age 75), and 60% of them were men (Figure 1B). The most common type of infection associated with CRE was the UTI, which accounted for 31.8% of all cases, followed by BSI or sepsis (~24%), pneumonia, (21.5%), and other infections (Figure 1C). Importantly, the mortality rate associated with CRE infections was lower than 5% in each of the reported years ~3.5%. (Figure 1D).

### 2.2. Microbiological Characteristics of CRE Infections 

**Figure 2:**
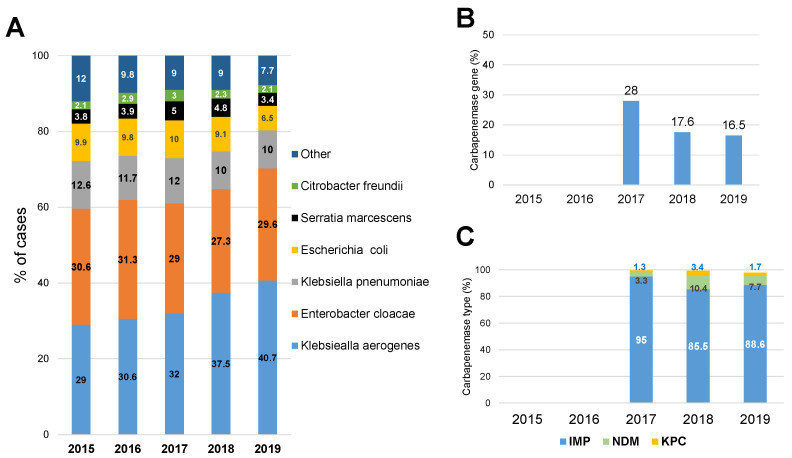
Key microbiological characteristics associated with CRE infections. (**A**) The most common bacterial strains resistant to carbapenems detected each year. (**B**) The percentage of bacterial strains that were confirmed to carry the carbapenemase gene. (**C**) The carbapenemase types that were identified each year. Data are shown as percentages. In figures (**B**,**C**), data from the years 2016 and 2017 are missing.

Contrary to the reported in other countries (developed and developing countries), where the most common CRE pathogens reported are *Klebsiella pneumonia* and *Escherichia coli*, in Japan, about 60% of CRE infections documented during this surveillance period were caused by *Enterobacter cloacae* and *Klebsiella aerogenes* (previously known as *Enterobacter aerogenes*), with *Enterobacter cloacae* being the most common pathogen isolated (~30%) in the first two years of CRE surveillance (2015 and 2016), and *Klebsiella aerogenes* (~40%) becoming the leading CRE pathogen isolated since 2017 (Figure 2A). Importantly, the presence of genes encoding carbapenemases was documented in less than 50% of the CRE bacterial strains isolated (Figure 2B), and, when present, the most common carbapenemase detected was by far the IMP type (active against imipenem) carbapenemase, which accounted for nearly 90% of cases, while a small fraction (~7%) of CRE strains harbor the NDM type (New Delhi Metallo-beta-lactamase) carbapenemase (Figure 2C). Unfortunately, we were not able to retrieve the data on carbapenemase type and the frequency of the carbapenemase gene in CRE strains isolated in 2015 and 2016. Interestingly, an analysis of CRE infections acquired during overseas travel in 2017 (*n* = 13) and 2018 (*n* = 42) showed that *Escherichia coli* (67%) and *Klebsiella pneumoniae* (16%) were the most commonly isolated strains (https://www.niid.go.jp/niid/ja/cre-m/cre-iasrd/9125-475d02.html, accessed on 19 May 2022). 

## 3. Discussion

Because they can cause difficult-to-treat infections and can spread efficiently in both the healthcare environment and the community setting, CREs have become a public health threat worldwide. Thus, the implementation of coordinated efforts at the local and international level is required to prevent the transmission of these pathogens. This communication summarizes important epidemiological aspects of CRE infections in Japan during the period 2015 to 2019. The annual number of reported CRE infections has remained relatively stable, with a tendency to increase in the last two years. Importantly, about 60% of infections are caused by both *Enterobacter cloacae* and *Klebsiella aerogenes*, the former being the most common pathogen in 2015 and 2016 (~30%), and the latter the leading pathogen since 2017 (~40%). The most common carbapenemase isolated in Japan was the IMP type, which is an Ambler class B carbapenemase that belongs to the metallo-β-lactamase group. These findings contrast with most publications from other regions of the world, where the most common carbapenemase isolated is *Klebsiella pneumoniae* carbapenemase (KPC), followed by D β-lactamase OXA-48 (OXA-48), and NDM [6]. 

Colonization with CRE pathogens, which implies that the bacteria can be isolated from individuals in the absence of clinical symptoms, is a prerequisite for infection, and since individuals colonized with CRE can spread the pathogen to other patients, determining the prevalence of colonization is critical for the management and prevention of CRE infections [7,8]. Since 2014, CRE infections have been systematically reported and registered in Japan under the national surveillance system. However, only those cases with clinical manifestations are eligible for notification, and, therefore, the prevalence of colonization with CRE pathogens is currently unknown. 

Although the precise worldwide prevalence of CRE is currently unknown due to the lack of data on bacterial resistance in many regions of the world, high prevalence has been reported in Europe, Brazil, China, Colombia, the United States, and India [9]. Contrary to the data reported in these countries, where the predominant CRE strain isolated is *Klebsiella pneumoniae*, followed by *Escherichia coli* [6,9], over the five year period, *Klebsiella aerogenes* and *Enterobacter cloacae* were the most common bacteria isolated in Japan. *Klebsiella aerogenes* was previously known as *Enterobacter aerogenes*, but comparative bacterial phylogenetics analysis showed this strain to be more closely related to *Klebsiella pneumoniae* than to the *Enterobacter* species [10]. Both *Klebsiella aerogenes* and *Enterobacter cloacae* are often associated with nosocomial infections, especially in patients exposed to invasive devices or procedures. A recent study showed that BSIs caused by *Enterobacter cloacae* and *Klebsiella pneumoniae* have similar clinical characteristics and prognoses, although a higher rate of co-morbidities was noticed among patients infected with *Enterobacter cloacae*, while those infected with *Klebsiella aerogenes* had a higher rate of previous antibiotic use [11]. Another study reported poor clinical outcomes, including death before discharge, and recurrent BSIs among patients infected with *Klebsiella aerogenes*, compared with those infected with other *Enterobacter species* [12]. The reason for these unique epidemiological features of CRE infection in Japan, including the carbapenemases produced by CPE and the predominant bacterial species, is currently unknown. Further studies are needed to better understand the epidemiology of CRE infections in Japan. Of particular interest is determining the prevalence of colonization with *Enterobacter cloacae* and *Klebsiella aerogenes* carrying carbapenemase in the nosocomial setting, not only among hospitalized patients but also among the healthcare workers and in the hospital environment. 

The reason for the higher incidence of CRE infections in males in Japan is also unknown. A large study from China reported that hospital-acquired infections caused by MDR bacteria were more frequent in males (64%) than in female patients (36%) [13]. Similarly, a study conducted in Korea showed that while community-acquired CRE infections were more common in women (61%), most patients (70%) with healthcare-acquired CRE infections were men [14]. In line with these observations, in a previous study conducted in Japan, the majority of patients with CRE infections were also male (~70%) [15]. It must be noted, however, that other studies conducted in Africa and Vietnam showed that CRE infections presented with similar frequencies among men and women [16,17]. 

Mortality rates associated with CRE infections depend largely on the site of infection, the underlying condition of the affected patients, and the availability of alternative antimicrobial therapies [13]. In this study, we found that, although most infections (>80%) were reported among individuals older than 65 years, mortality rates were generally lower than 5% in each reported year, which is lower than reported in other studies, with some reports showing mortality rates higher than 50%, especially among hospitalized patients with BSI and sepsis [18,19]. These disparate observations are intriguing, but the exact mechanism behind the lower mortality associated with CRE infections in Japan is currently unknown. A possible explanation could be associated with the CRE bacteria predominant in Japan (*Klebsiella aerogenes* and *Enterobacter cloacae*), which may cause less severe infections compared to *Escherichia coli* and *Klebsiella pneumonia*, as occurs abroad. Additionally, whereas CRE types found overseas are often multi-drug-resistant, it seems that CRE pathogens isolated in Japan tend to be sensitive to other antibacterial drugs, which permits the utilization of alternative antimicrobial options to treat these infections. Data from a previous study conducted in Japan may also provide some clues. In that study, factors associated with increased risk for 28 day mortality in patients with CRE infections were mechanical ventilation, solid metastatic cancers, and BSI [15]. The fact that in 75% of patients the reported CRE infections do not associate with BSI may account for the relative lower mortality associated with CRE infections in Japan. Further studies are needed to clarify these issues.

In conclusion, the present study revealed unique epidemiological characteristics associated with CRE infections in Japan. Over a period of five years, *Klebsiella aerogenes* and *Enterobacter cloacae* were the most common bacterial strains associated with CRE infections. Lower mortality rates, relative to overseas data, were also reported among patients with CRE infections. Further studies are needed to determine the prevalence of CRE colonization, especially in the healthcare setting, and to elucidate the mechanisms behind the predominance of *Klebsiella aerogenes* and *Enterobacter cloacae*.

## 4. Materials and Methods

### Data Management and Analysis 

Disease rates were abstracted from nationwide statistics obtained from the publicly available dataset and from annual surveillance reports from the National Institute of Infectious Diseases (NIID) of Japan. Data management, and statistical analyses and figures, were performed using Excel (version 2016) software package (Microsoft Corporation, Redmond, WA, USA). For CRE identification, a drug susceptibility test was conducted, and the presence of CRE isolates was determined according to the criteria issued by the Ministry of Health, Labour, and Welfare (MHLW) of Japan, which implies the presence of *Enterobacteriaceae* isolates with either MEPM-MIC ≥2 µg/mL or IPM-MIC ≥2 µg/mL and CMZ-MIC ≥64 µg/mL. Further tests included the screening for β-lactamase production by disk diffusion, and the detection of carbapenemase genes (KPC-type, NDM-type, VIM-type, IMP-type, and OXA-48-type) by PCR using specific primers.

## Data Availability

Not applicable.
